# Geometrically pinned magnetic domain wall for multi-bit per cell storage memory

**DOI:** 10.1038/srep28590

**Published:** 2016-06-23

**Authors:** M. Al Bahri, R. Sbiaa

**Affiliations:** 1 Department of Physics, Sultan Qaboos University, P.O. Box 36, PC 123, Muscat, Oman

## Abstract

Spintronic devices currently rely on magnetic switching or controlled motion of domain walls (DWs) by an external magnetic field or a spin-polarized current. Controlling the position of DW is essential for defining the state/information in a magnetic memory. During the process of nanowire fabrication, creating an off-set of two parts of the device could help to pin DW at a precise position. Micromagnetic simulation conducted on in-plane magnetic anisotropy materials shows the effectiveness of the proposed design for pinning DW at the nanoconstriction region. The critical current for moving DW from one state to the other is strongly dependent on nanoconstricted region (width and length) and the magnetic properties of the material. The DW speed which is essential for fast writing of the data could reach values in the range of hundreds m/s. Furthermore, evidence of multi-bit per cell memory is demonstrated via a magnetic nanowire with more than one constriction.

A magnetic domain wall (DW) is a spatially localized change of magnetization configuration in a ferromagnetic material. The motion of DW using spin transfer torque (STT) has attracted great interest in fundamental theoretical studies and promising potential applications, such as high density magnetic storage and logic devices^1^,^2^,^3^,^4^,^5^,^6^,^7^,^8^,^9^,^10^,^11^. For memory application, several requirements need to be fulfilled for a good functionality. For instance, the non-volatility is desirable for saving the power consumption while scaling down the device size; i.e. increasing the storage capacity, requires low writing and reading currents^12^,^13^. Magnetic tunnel junction (MTJ) where two ferromagnetic layers separated by a tunnel barrier was the first prototype of magnetic memory devices. Writing and erasing the data on MTJ memory could be done by a polarized current through reversal of the magnetization of a soft layer (called memory layer). Although many of the requirements above can be achieved in an MTJ^14^,^15^,^16^,^17^,^18^,^19^,^20^, the limitation to two states remains an obstacle toward high capacity memory. Multi-level MRAM where two memory layers could be used to store four states was proposed to boost the storage capacity^21^,^22^. Storing even larger data in one cell is possible by moving DW at different positions within the nanowire. In DW-based memory, the stability and speed of DW have to be controlled and optimized for actual application. Stabilizing DW at desired positions is very important for a good functionality of the storage memory. Although many reports were devoted to study domain wall dynamics and its motion under magnetic field, electric field and/or polarized current^23^,^24^,^25^,^26^,^27^,^28^,^29^,^30^,^31^,^32^,^33^,^34^, controlling its position and its stability remains a big challenge^35^,^36^,^37^,^38^,^39^,^40^. Creating artificial defects were proposed and investigated to generate a potential that acts as a pinning site for DW^35^,^36^. Designing pinning sites by lithography is challenging since this requires a high resolution process that is much better than making the nanowire itself. It is much easier to create notches when the nanowire dimension are in the hundreds nanometers and above. However, scaling down the nanowire size to few tens of nanometer with even smaller notches is a tremendous technological challenge. In this work, we propose a new way for pinning DW in magnetic nanowire with adjustable size and position. The method is based on designing portions of a magnetic nanowire with the same size but with a small off-set in either one direction or both. Figure 1(a) shows a proposed nanowire with a single step. For device fabrication, creating notches on a nanowire for pinning DW requires additional nanofabrication process after the nanowire is made. Furthermore, since the dimension of the notches have to be smaller than the width of the nanowire, their positions and size uniformity will be challenging and could be a serious obstacle for their implementation. In our proposed scheme shown in Fig. 1(a), the design could be made in a single process. It is also easy to create an off-set of the two small patterns in either *x* or *y* directions prior to the beam exposure on the resist (polymer) which serves as an etching mask. The study demonstrates that DW could be stabilized in the stepped region (constriction) and the pinning current depends on its dimension (step depth *d* and step length *l*) and the magnetic material properties such as anisotropy energy *K*_u_ and saturation magnetization *M*_s_. Figure 1(b) shows the case of multi-step device for multi-bit per cell magnetic memory device.

## Results

**Figure 1:**
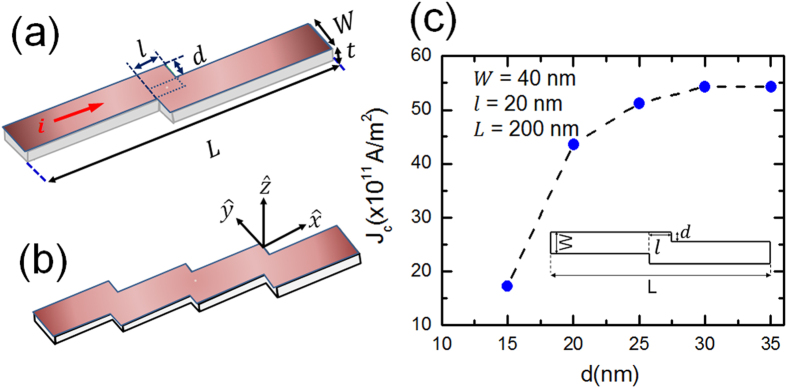
(**a**) Schematic representation of a stepped type nanowire where the step can be used for pinning domain wall. The dimensions of the nanowire and the step are shown in the figure. The magnetization is aligned in the film plane with easy axis along the *x*-axis. (**b**) An extended design to multi-bit per cell magnetic memory. (**c**) the critical current *J*_c_ to move domain wall from the pinning region as a function of the step depth *d* which is the off-set in the *y*-axis direction. The nanowire length *L* and width *W* were fixed to 200 nm and 40 nm, respectively and the magnetic properties of the investigated material are *M*_s_ = 600 kA/m and *K*_u_ = 1.0 × 10^5^ J/m^3^.

We consider a magnetic nanowire of length *L*, width *W*, and thickness *t* with a stepped region. As the objective was to stabilize DW in this region we also considered the nanowire is made of two parts with an off-set *l* in the *x* direction and *d* in the *y* direction as illustrated in Fig. 1(a).

Micromagnetic simulation was conducted to study the magnetic DW motions in the nanowire with the proposed scheme [http://math.nist.gov/oommf]. A magnetic material with in-plane anisotropy was considered in this work with a mesh size of 2.5 nm × 2.5 nm × 3 nm. The polarized electric current was flowing along the nanowire in the positive *x* direction and the magnetization was initially aligned in the opposite direction. In the first part of this study, the effect of stepped region dimension *l* and *d* on DW dynamics is investigated while in a second part, we were interested in the correlation between the magnetic properties and DW stability. In all this study, the length *L*, the width *W* and the thickness *t* were fixed to 200 nm, 40 nm and 3 nm, respectively. Also the exchange stiffness *A* and damping constant *α* of the material were fixed to 1.0 × 10^−11^ J/m and 0.05, respectively. These values are typical for materials such as Co, CoFe or CoFeB alloys.

### Nanoconstriction dimension and DW dynamics

**Figure 2:**
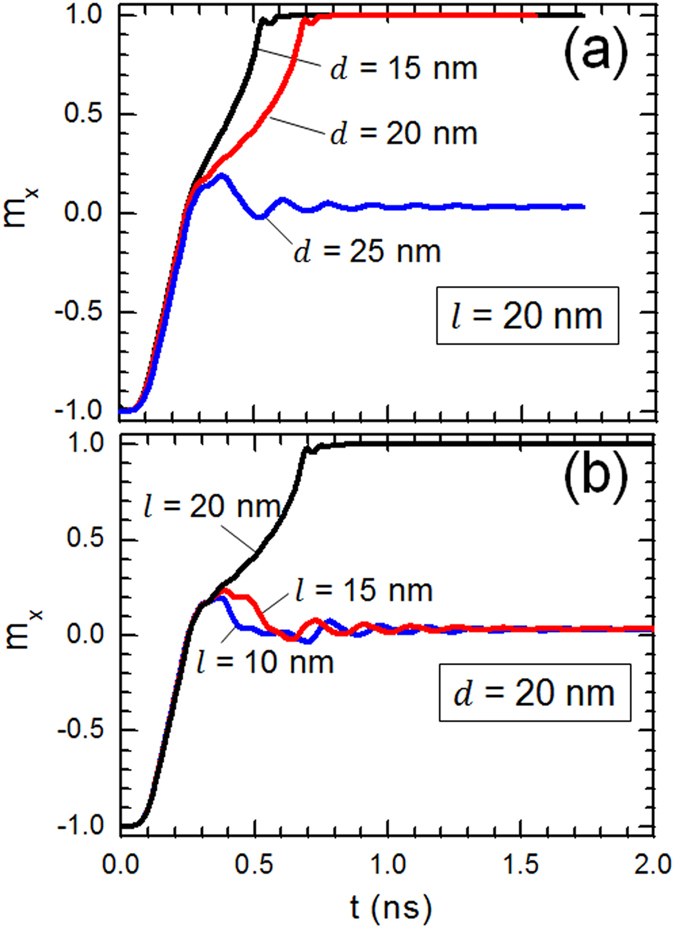
Normalized *x*-component of nanowire magnetization as a function of time for different values of the step depth *d*. The length and width of the nanowire were fixed to 200 nm and 40 nm, respectively. The magnetic properties of the investigated material are *M*_s_ = 600 kA/m and *K*_u_ = 0.5 × 10^5^ J/m^3^ and *J* = 4.84 × 10^12^ A/m^2^.

By varying *d* and *l* we were able to stabilize DW at the stepped region for current density below a critical value *J*_c_. For instance, it can be seen from Fig. 1(c) that at *l* = 20 nm, it is not possible to stabilize DW for values of *d* smaller than 15 nm which means that there are optimal dimensions of the stepped region to favour DW stability. For current density values above *J*_c_, a continuous movement of DW from one side to the other was observed. These calculations were carried out for material with *M*_s_ = 600 kA/m and *K*_u_ = 1.0 × 10^5^ J/m^3^. Figure 2 presents plots of the time dependence of the normalized *x*-component of magnetization *m*_x_ = *M*_x_/*M*_s_ for several values of *d* and *l* and for an applied current density *J* = 4.8 × 10^12^ A/m^2^. It can be noticed from Fig. 2(a) that the stabilization of DW occurs for *d* > 25 nm, i.e. larger applied current requires larger *d* for DW pinning. Similarly, the time dependence of *m*_x_ for *d* = 20 nm and different values of *l* is shown in Fig. 2(b). This means that stabilizing DW within the vicinity of stepped region is possible by selecting the optimal values of *d* and *l* for each applied current density. It is worthy to note that for large *d* [Fig. 2(a)] or small *l* [Fig.2(b)], the velocity of DW motion is reduced and for the optimal values of *d* and *l* it starts to oscillate before the pinning occurs.

**Figure 3:**
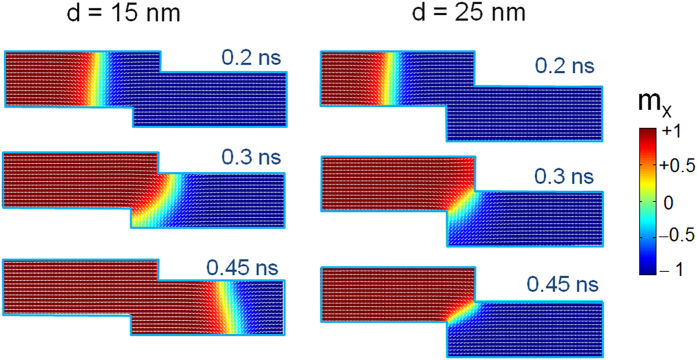
Different positions for domain walls at different times for two values of *d*. The length and width of the nanowire were fixed to 200 nm and 40 nm, respectively. The magnetic properties of the investigated material are *M*_s_ = 600 kA/m and *K*_u_ = 0.5 × 10^5^ J/m^3^.

To elucidate the effect of device geometry on DW stability and its dynamics, the magnetic configuration of a moving DW for two values of step depth *d* was examined. The current density and length of the step *l* were fixed to 4.84 × 10^12^ A/m^2^ and 20 nm, respectively (Fig. 3). The snapshot images were taken at three different positions within the nanowire. For *t* = 0.2 ns, DW is still at the first half of the nanowire and did not reach the stepped area. For the case of a device with *d* = 25 nm, DW remained pinned at the stepped region while for *d* = 15 nm, it moved continuously as can be seen for *t* = 0.45 ns. The same result can also be observed for *d* = 20 nm case. It can be noticed that under the conditions discussed in Figs 2 and 3, we observed a clear transverse type DW.

### Material properties and DW dynamics

**Figure 4:**
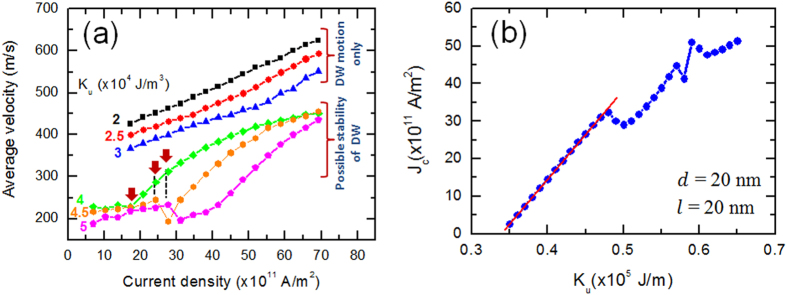
(**a**) Domain wall average velocity varies with current density for different values of magnetic anisotropy energy. The nanowire length *L* and width *W* were fixed to 200 nm and 40 nm, respectively and the saturation magnetization of the investigated material is *M*_s_ = 600 kA/m. (**b**) The relationship between critical current density and anisotropy energy.

In the following, the effect of magnetic properties of the material on DW dynamics for a fixed device geometry (*L* = 200 nm, *W* = 40, *l* = 20 nm and *d* = 20 nm) will be presented. First, we varied *K*_u_ as reported in Fig. 4 while *M*_s_ was fixed to 600 kA/m. The average velocity taken from time dependence of *m*_x_ is plotted as a function of *J* for several values of *K*_u_ [Fig. 4a)]. For low anisotropy materials (*K*_u_ < 3.5 × 10^4^ J/m^3^), no DW could be stabilized and we observed only DW movement with an almost linear behavior of *v* with *J*. For *K*_u_ = 2 × 10^4^ J/m^3^, DW moves freely without being affected by the change in the nanowire geometry as reported by Zhang *et al*.^41^. The velocity can be expressed by:




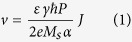




where *ħ* is the reduced Planck constant, *e* is charge of electron, *γ* is the gyromagnetic ratio, *P* is the spin polarization of the current and *ε* is the non-adiabatic parameter^42^. It is worthy to note that for *K*_u_ = 3 × 10^4^ J/m^3^, a deviation from a linear behavior was clearly seen. This is because although DW could not be stabilized at the pinning region, it takes some time to be released.

We initially started with all the magnetic moments aligned in the negative *x* direction and it takes some time to observe a creation of DW under spin transfer torque effect. The average velocity is calculated from the time the DW is created until it is pinned at the stepped region (for large *K*_u_ values) or vanishes at the end of the nanowire (for small *K*_u_ values).

**Figure 5:**
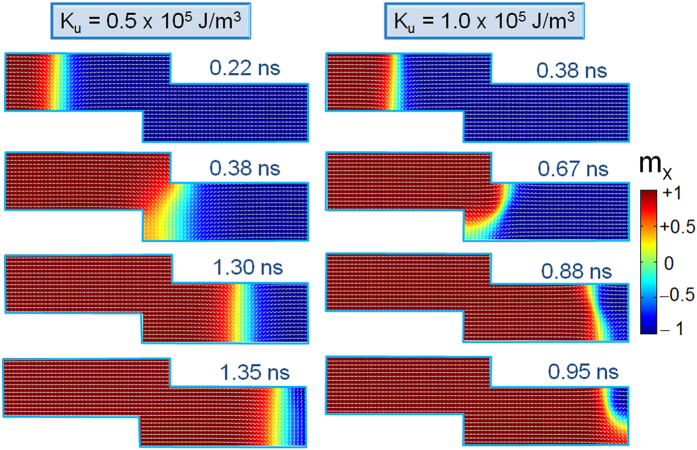
Snapshot images of the nanowire with constriction for *K*_u_ = 0.5 × 10^5^ J/m^3^ and 1.0 × 10^5^ J/m^3^. The length and width of the nanowire were fixed to 200 nm and 40 nm, respectively and the stepped region dimension *d* and *l* were both fixed to 20 nm. The calculation was carried at *M*_s_ = 600 kA/m and *J* = 5.5 × 10^12^ A/m^2^.

**Figure 6:**
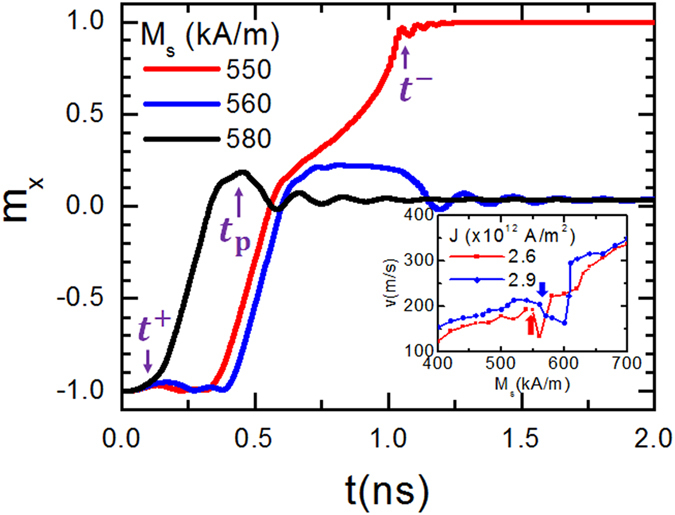
Normalized *x*-component of nanowire magnetization as a function of time for different values of *M*_s_. The device lateral dimensions are *L* = 200 nm, W = 40 nm, *l* = 20 nm and *d* = 20 nm. The material magnetic anisotropy is *K*_u_ = 0.5 × 10^5^ J/m^3^ and *J* = 2.6 × 10^12^ A/m^2^.

**Figure 7:**
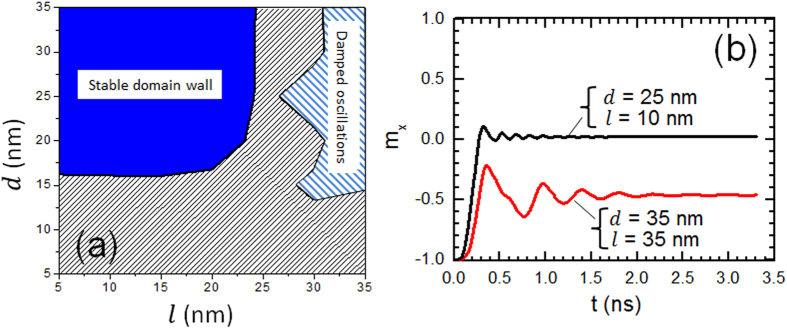
(**a**) Phase diagram *d*–*l* for device with lateral dimension *L* = 200 nm and *W* = 40 nm. The material magnetic anisotropy are *M*_s_ = 600 kA/m and *K*_u_ = 0.5 × 10^5^ J/m^3^. (**b**) Normalized *x*-component of nanowire magnetization as a function of time for selected values of *d* and *l*.

As *K*_u_ increases (*K*_u_ ≥ 3.5 × 10^4^ J/m^3^), a pinned DW could be observed in the middle of nanowire for *J* < *J*_c_ which is indicated by the arrow in Fig. 4(a). This transition from a stable to unstable DW is accompanied by a drop in the velocity. This is mainly due to an increase of the time DW takes to be released from the stepped region. For further increase of *J*, a steady increase of *v* is revealed. After plotting *J*_c_ as a function of *K*_u_ when DW stability is possible [Fig. 4(b)], we noticed that there is a region where *J*_c_ increases linearly with *K*_u_ (*K*_u_ between 3.5 × 10^4^ J/m^3^ and 4.75 × 10^4^ J/m^3^). However, as *K*_u_ becomes larger, more complex behavior of magnetic domains is observed. By looking at the details of magnetic moments configuration, for two values of *K*_u_, the shape of DW and its evolvement with time could be imaged as shown in Fig. 5. The DW position within the left side of the nanowire is not shown for simplicity. When DW is created and until it reaches the stepped region we observed a movement of a transverse DW for both values of *K*_u_. However, as the DW passed the stepped region, a change in DW configuration was revealed. For *K*_u_ = 0.5 × 10^5^ J/m^3^, the transverse type DW could still be seen until it vanishes at the end of the nanowire. In contrast, for *K*_u_ = 1.0 × 10^5^ J/m^3^, DW starts to bend and an antivortex type DW could be observed. A current density *J* = 5.5 × 10^12^ A/m^2^ was used in this calculation and snapshot images were taken times corresponding to desired DW locations based on *m*_x_ versus time graph. It is noticed that antivortex type DW moves faster than transverse type^43^. Similar to the study conducted on *K*_u_ effect on DW dynamics shown above (Figs 4 and 5), it was observed that there is a minimum *M*_s_ value for stabilizing DW at the stepped region. Figure 6 is a plot of *m*_x_ versus time for different values of *M*_s_ and *K*_u_ = 0.5 × 10^5^ J/m^3^. The current density was fixed to 2.6 × 10^12^ A/m^2^ and the device length and width were kept same as reported in Fig. 5. No pinning of DW could be seen for *M*_s_ smaller than 560 kA/m. Neverthless, the slope in time dependence curve of *m*_x_ is an indication of a change in the DW velocity; i.e, DW moves with a slower speed after passing the constriction region. For *M*_s_ > 550 kA/m, DW could be stabilized at the center as shown in Fig. 6 for *M*_s_ = 560 and 580 kA/m. This is an important finding of this study. It is possible to stabilize DW by creating an off-set of the nanowire at desired position. Furthermore, material with larger *M*_s_ favors a faster DW creation for given current density as indicated by *t*^+^ for 580 kA/m case. To evaluate the velocity of DW, we considered the time difference between *t*^+^ and the time when DW is either pinned *t*_p_ (large *M*_s_ case) or annihiled *t*^−^ (low *M*_s_ case). In the insert of Fig. 6, the velocity *v* of DW was plotted as a function of *M*_s_ for *J* of 2.6 × 10^12^ A/m^2^ and 2.9 × 10^12^ A/m^2^. A continuous increase of *v* with *M*_s_ is observed. The bold arrows show the critical *M*_s_ separating non-pinned and pinned DW ranges. This critical value depends on the current density, device dimension and *K*_u_. It is important to mention about the relatively large values of DW velocity obtained from the time dependence of *m*_x_ which is beneficial for a fast writing of data by a polarized electric current. For a good stability of DW in the proposed device, it is important to optimize the values of *d* and *l*. Figure 7 displays the calculated phase diagram for nanowire with *M*_s_ = 600 kA/m and *K*_u_ = 0.5 × 10^5^ J/m^3^. The stability of DW inside the nanowire could be seen for *d* larger than 15 nm and *l* below 25 nm (W was fixed to 40 nm). We also observed damped oscillation for large value of *d* and *l* as shown in dashed region of Fig. 7. DW could be stabilized in very narrow range of current density. More interestingly, DW with large amplitude oscillation could be seen in this range.

**Figure 8:**
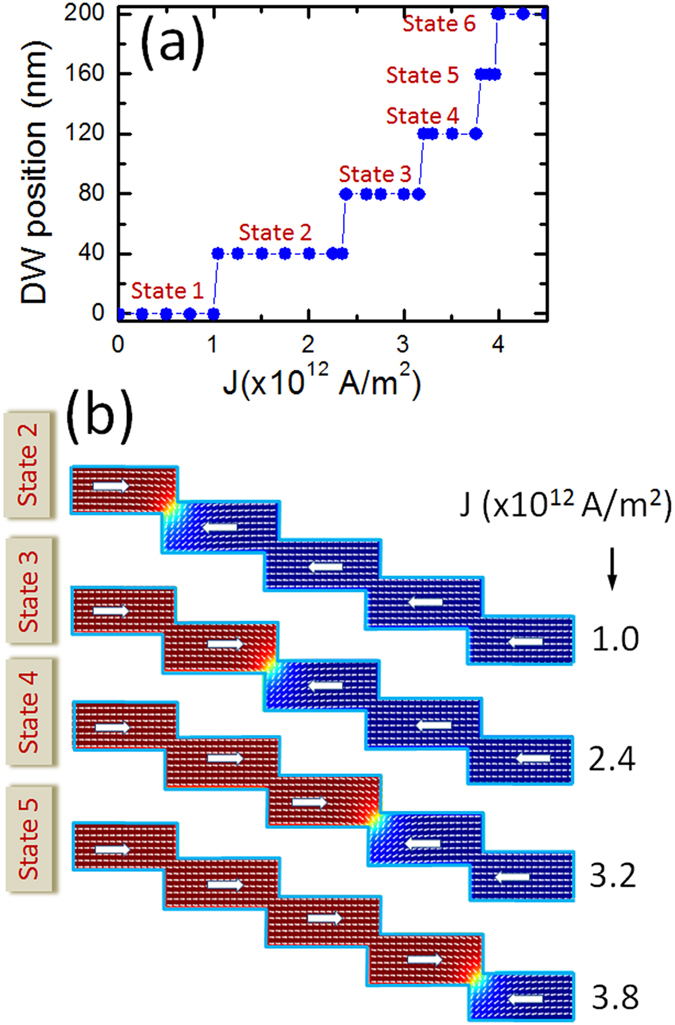
(**a**) The six states of nanowire of dimentions *L* = 200 nm, *W* = 40 nm, *l* = 10 nm and *d* = 30 nm. The magnetic properties of the investigated material are *M*_s_ = 600 kA/m and *K*_u_ = 0.5 × 10^5^ J/m^3^. (**b**) Snapshot images of four states obtained at different current density values.

For a good performance of the memory device, it is also important to store more than 2 bits/cell (i.e. four states) as experimentally demonstrated in current-perpendicular to plane magnetoresistive devices based on magnetization switching^21^. For this purpose, we investigated the possibility of storing six states using a nanowire with four constricted regions. The device lateral dimensions are *L* = 200 nm, *W* = 40 nm, *l* = 10 nm and *d* = 30 nm. Figure 8(a) is a plot of DW position for different current density values. A transition from state 1, where all spins of the device are aligned in the −*x* direction, to state 2 where only the spins on the left side of the first nanoconstriction are reversed, occurs at *J* = 1.0 × 10^12^ A/m^2^. The second transition, i.e. from state 2 to state 3, happens at 2.4 × 10^12^ A/m^2^ as shown in Fig. 8. The six states obtained are very stable for the device dimension reported above. For clarity, states 1 and 6 are not shown. We conducted calculations with same values of *M*_s_, *K*_u_, *L* and *W*, except *l* and *d* which were fixed both to 20 nm as used is Fig. 6 but we were not able to obtain all the six states shown in Fig. 8(b).

## Discussion

We have demonstrated that in magnetic nanowire with a stepped region, DW could be precisely pinned. The depinning current density could be easily adjusted by the constriction dimension and the materials properties. As *K*_u_ increases, larger *J*_c_ is required to move a transverse type DW from one state to the other. However, further increase of *K*_u_ leads to an antivortex type DW with a lower velocity. Similarly, a resonably large *M*_s_ is needed to pin DW. Its velocity is improved as *M*_s_ increases. The proposed scheme was extended to multi-step device which showed a clear stability for DW at different positions. The magnitude of DW depinning current and its movement speed could be well tailored by adjusting the gerometry of the device and the materials properties. Optimal values for *K*_u_ and *M*_s_ are required for each device.

## Methods

We investigated the magnetization dynamics of a stepped nanowire with micromagnetic simulations. The simulations are performed with the object-oriented micromagnetic framework (OOMMF) which was extended to consider the current-induced magnetization dynamics as described by the Landau-Lifshitz-Gilbert equation with additional spin-transfer torque terms:




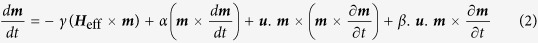




where ***m*** is the local normalized magnetization, *γ* the gyromagnetic ratio, ***H***_eff_ the effective field, *α* the Gilbert damping factor, and *β* the nonadiabatic spin-transfer parameter^41^,^44^.

The local effective magnetic field ***H***_eff_ includes the exchange, anisotropy and magnetostatic fields. The vector ***u*** is the adiabatic spin torque which has the dimension of velocity and is proportional to the current density according to 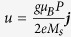


where ***j*** is the current density, *g* is the Lande factor, μ_B_ the Bohr magnetron (μ_B_ = 0.927 × 10^−20^ emu), *e* the electron charge, *P* the polarization rate of the current fixed to 0.6 and the nonadiabatic spin-transfer parameter *β* to 0.02.

## Additional Information

**How to cite this article**: Bahri, M. A. and Sbiaa, R. Geometrically pinned magnetic domain wall for multi-bit per cell storage memory. *Sci. Rep.* **6**, 28590; doi: 10.1038/srep28590 (2016).
